# The Cytoskeletal Protein RHAMM and ERK1/2 Activity Maintain the Pluripotency of Murine Embryonic Stem Cells

**DOI:** 10.1371/journal.pone.0073548

**Published:** 2013-09-03

**Authors:** Jihong Jiang, Pooja Mohan, Christopher A. Maxwell

**Affiliations:** Department of Pediatrics, Child & Family Research Institute, University of British Columbia, Vancouver, British Columbia, Canada; Baylor College of Medicine, United States of America

## Abstract

Receptor for hyaluronan mediated motility (RHAMM, encoded by *HMMR*) may be a cell-surface receptor for hyaluronan that regulates embryonic stem cell pluripotency and differentiation, however, a precise mechanism for its action is not known. We examined murine embryonic stem cells with and without hemizygous genomic mutation of *Hmmr*/RHAMM, but we were not able to find RHAMM on the cell-surface. Rather, RHAMM localized to the microtubule cytoskeleton and along mitotic spindles. Genomic loss of *Hmmr*/RHAMM did not alter cell cycle progression but augmented differentiation and attenuated pluripotency in murine embryonic stem cells. Through a candidate screen of small-molecule kinase inhibitors, we identified ERK1/2 and aurora kinase A as barrier kinases whose inhibition was sufficient to rescue pluripotency in RHAMM^+/-^ murine embryonic stem cells. Thus, RHAMM is not found on the cell-surface of embryonic stem cells, but it is required to maintain pluripotency and its dominant mechanism of action is through the modulation of signal transduction pathways at microtubules.

## Introduction

Hyaluronan (HA) is an extracellular polysaccharide, and HA-rich hydrogels maintain human embryonic stem (ES) cells in their undifferentiated state [1]. Extracellular receptors for HA are likely responsible for the transmission of intracellular signals that maintain ES cell pluripotency. Indeed, CD44, the major cellular receptor for HA, is critical to the expansion, differentiation, and pluripotency of a wide range of stem cells, including cancer stem cells [2], neural [3], mesenchymal [4] and epidermal stem cells [5]. Moreover, human ES cells also express receptor for hyaluronan mediated motility (RHAMM, encoded by *HMMR*) [6], a putative and controversial receptor for HA [7]. The expression of RHAMM is dramatically reduced with differentiation *in vitro*, and the silencing of RHAMM with siRNA disrupts the self-renewal of human ES cells [6]. Thus, the extracellular localization of RHAMM, and its putative engagement of HA, may be a critical regulatory cue that decides the cellular fate of ES cells; such an action for RHAMM would be vital to multiple aspects of stem cell biology, including stem cell niches, tissue engineering, and biomaterials.

Originally identified as a peptide in the supernatants from proliferative fibroblasts [8], RHAMM may be passively released to the extracellular space through the cell death that accompanies pathological states, such as cancer and inflammation [9]. However, RHAMM is an intracellular protein that associates with both microtubules and actin microfilaments [10]. RHAMM forms a complex with the dynein molecular motor to localize to the centrosome [11] and enable mitotic spindle assembly [12–14], and RHAMM regulates signal transduction at microtubules, including the aurora kinase A (AURKA) [14] and ERK1/2 [15] pathways. Thus, the putative physiological role is not yet clear for RHAMM during the self-renewal of ES cells.

Here, we examine the localization and action of RHAMM within mouse ES cells, which contain an insertional mutation in one allele of the *Hmmr*/RHAMM gene. We do not find that RHAMM is expressed on the cell-surface but rather that RHAMM is a cytoskeletal and mitotic spindle protein in these cells. The hemizygous genomic loss of *Hmmr*/RHAMM attenuates mouse ES cell pluripotency and we use a small-molecule screen to discover that the phosphorylation of ERK1/2 and AURKA are elevated and these kinases act as barriers to pluripotency in RHAMM^+/-^ mouse ES cells.

## Materials and Methods

### Mouse ES cell Culture

RHAMM^+/-^ mouse ES cell-lines (BB0166- MMRRC: 026467-UCD; and XP0038- MMRRC: 028514-UCD) with mutational inactivation of one allele for the *Hmmr/*RHAMM gene and the parental control RHAMM^+/+^ mouse ES cell-line (*E14*TG2a) were purchased from the International Gene Trap Consortium through a Mutant Mouse Regional Resource Center (University of California, Davis). Briefly, mouse ES cells were maintained on mitomycin C-treated mouse embryonic fibroblast (MEF) cells in medium consisting of high-glucose Dulbecco’s modified Eagle’s medium (DMEM) supplemented with fetal bovine serum (FBS) 16% (ES-qualified, Invitrogen), L-glutamine 2 mM, non-essential amino acids (NEAA) 0.1 mM, leukemia inhibitory factor (LIF) 1000 U/ml (Chemicon), and 2-mercaptoethanol 143 µM. For feeder-free cultures, mouse ES cells were grown on CellBind plates (Corning) in medium consisting of Glasgow minimal essential medium (GMEM) (Sigma) containing FBS 15% (ES-qualified, Invitrogen), L-glutamine 2 mM, sodium pyruvate 1 mM, NEAA 0.1 mM, LIF 1000 U/ml, and 2-mercaptoethanol 66 µM.

### Quantitative Reverse Transcriptase Polymerase Chain Reaction (RT-PCR)

Total RNA was extracted from cells with a RNeasy mini kit (Qiagen), treated with DNase I (Invitrogen), and converted to cDNA with a high capacity cDNA RT kit (Applied Biosystems). Real-time quantitative RT-PCR were performed as described [16] using the primers listed in Table S1.

### Immunostaining

Cells were grown on coverslips coated with 0.1% gelatin, fixed with 4% paraformaldehyde (PFA) for 15 minutes (min) at room temperature (RT). Cells were permeablized and blocked with 0.3% triton X-100, 10% normal donkey serum, 0.1% BSA in PBS, and incubated with antibody listed in Table S2 at their respective dilutions. For staining the antigen on the cell surface, detergent was omitted from the buffer.

Following three washes in PBST, the following secondary antibodies were applied for 1 hour at RT: anti-mouse Alexa-488, anti-mouse Alexa-594, anti-mouse Alexa-647, anti-rabbit Alexa-488, anti-rabbit Alexa-594, or anti-rabbit Alexa-647 (1:1500, Life Technologies). Coverslips were mounted with Prolong Gold antifade reagent (Life Technologies) and counterstained with DAPI. Confocal microscopy was performed using a FluoView Fv10i confocal laser scanning microscope (Olympus) and images were processed with Olympus Fluoview software.

### Immunoblotting

Cells were lysed in RIPA buffer (25 mM Tris-HCl, pH7.4, 150 mM NaCl, 1% Triton X-100, 0.5% Na deoxycholate, 0.1% SDS) supplemented with Protease inhibitor (Roche) and 2 mM Na _3_VO_4_ and 50 mM NaF. Cell lysates were clarified by centrifugation at 16,000 X *g* for 10 min at 4^o^C, and protein concentration was determined by BCA protein assay kit (Thermal Scientific). Cell lysates were mixed with SDS sample buffer, separated by SDS-PAGE, and blotted with the following antibodies: anti-RHAMM (Epitomics), anti-Oct3/4 (StemCell Technologies), anti-NUMB (Abcam), anti-(p) ERK1/2 (Cell Signaling), anti-ERK (Cell Signaling), anti-AURKA (Abcam), anti-(p) AURKA (Cell Signaling), and anti-Actin (Sigma).

### Fluorescent Activated Cell Sorting Analysis

Mouse ES cells were harvested by trypsinization and washed. For cell surface protein expression, cells were incubated with the primary antibodies (30 min, 4^o^C), washed twice, and then incubated with PE-conjugated anti-mouse IgM (BD pharmingen) or anti-rabbit Alexa-647 IgG (30 min, 4^o^C) and washed twice. For intracellular protein expression, cells were fixed and permeabilized with fresh 4% PFA (15 min, RT) and methanol (10 mins, -20°C), or methanol alone, before immunostaining. The following primary antibodies were used in this study: anti-SSEA (StemCell Technologies), anti-N terminal RHAMM (Epitomics), anti-C terminal RHAMM (Epitomics), anti-TPX2 (Novus). For cell cycle analysis, cells were fixed with 70% ethanol at -20°C overnight, and then stained with 60 µg/ml propidium iodide (Invitrogen) for 30 min. FACS analysis was performed using a FACSCalibure2 flow cytometer (BD biosciences) and the CellQuest software.

### Cell proliferation

To measure the doubling time for mouse ES cell-lines, 10^5^ cells were seeded in 24 well CellBind plates. Cell numbers were counted 24, 48, 72 and 96 hours after plating. Doubling time was calculated by the equation *Yend = Ystart × 2* ^*(t/T)*^, where T is the doubling time, Ystart is the starting number of ES cells (24 hours after plating) and Yend is the ending number of ES cells after a period of time t.

### Alkaline Phosphatase (AP) Assays and Embryoid Body Formation

Cells were stained with alkaline phosphatase staining kit II (Stemgent) following the manufacture’s instruction. Images were acquired with a Zeiss Axiovert 200 M inverted microscope. Static suspension culture in knockout DMEM (Invitrogen) containing 15% knockout serum replacement (Invitrogen) on ultra low attachment culture dishes was utilized to form embryoid bodies from Rhamm^+/+^ and Rhamm^+/-^ ES cells.

### Inhibitor Screen and High Content Analysis

Mouse ES cells were plated at 2 x 10^4^ per well in CellBind 24 well plates without feeder cells and treated with indicated small molecular inhibitors for 3 days in triplicates. Medium with inhibitor was changed daily. The following inhibitor concentrations were used: 50 nM MLN8237 (AURKA inhibitor, Selleck chemicals), 4 µM PD184352 (inhibitor of the ERK1/2 pathway, Selleck chemicals), 10 µM U0126 (inhibitor of the MEK/ERK pathway, Cell signaling), 2 µM P38 inhibitor 202190 (Selleck chemicals), or 4 µM gamma-secretase inhibitor X (EMD).

Automatic high content analysis of ES cell colonies was done using an InCell analyzer (GE) or an ImageXpress Micro XL (Molecular Devices). Briefly, after AP staining, images were analyzed with InCell developer software or MetaXpress 5.0. The intensity of AP staining was inverted and ES cell colonies were segmented and categorized into different groups based on their shape factor and the density of AP staining.

### Statistical Analysis

Two-tailed pairwise Student’s *t* test was used to analyze results obtained from two samples with one time point. The results were considered significant at p < 0.05. FACS analysis was performed on at least 10,000 events per replicate after gating out cell debris and doublets based upon the forward and side scatter.

## Results

### RHAMM is a cytoskeletal protein and is not on the cell surface of mouse ES cells

**Figure 1:**
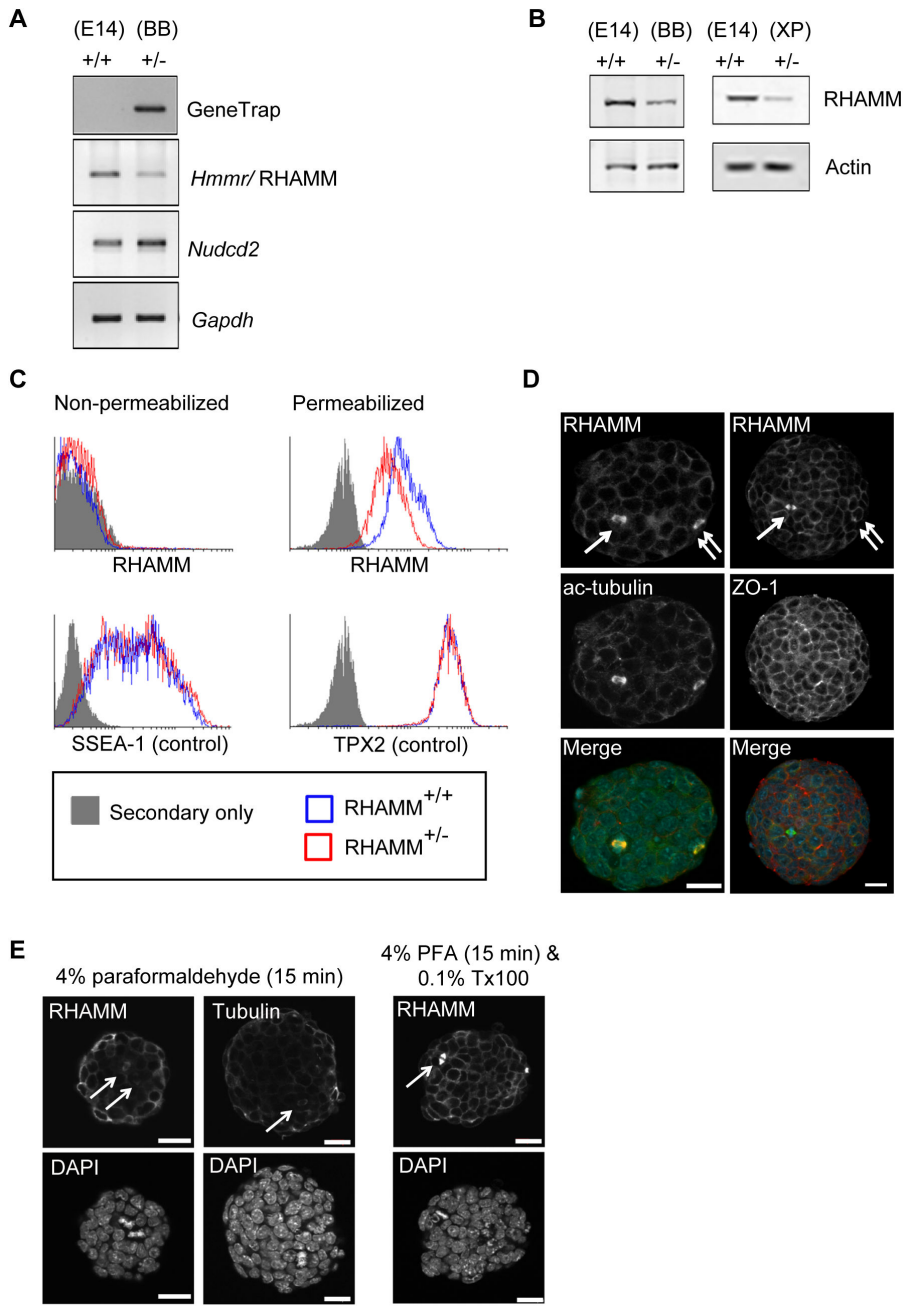
RHAMM is not a cell surface but an intracellular cytoskeletal protein in mouse embryonic stem (ES) cells. (A) Confirmation of the gene trap insertion and expression levels of RHAMM and Nudcd2 in parental (E14TG2; E14) and RHAMM^+/-^ (BB0166; BB) mouse ES cells were measured by RT-PCR. GAPDH served as an internal control. Gene expression of RHAMM was reduced in RHAMM^+/-^ cells whereas *Nudcd2* was unaffected by the gene trap insertion. (B) Western blot analysis revealed a marked reduction of RHAMM protein in RHAMM^+/-^ mouse ES cells (BB0166). Similar reduction of RHAMM was observed in an alternate RHAMM^+/-^ mouse ES cell-line, XP0038. Actin served as a loading control. (C) Fluorescence activated cell sorting (FACS) of non-permeabilized (left panel) mouse ES cells failed to detect extracellular RHAMM using a C-terminal directed antibody. Similar results were obtained with an N-terminal directed antibody (not shown). Relative to negative control secondary antibody only, both cell-lines were strongly positive for SSEA-1. Alcohol permeabilized mouse ES cells, however, were strongly positive for both RHAMM and the intracellular positive control protein TPX2 (right panel). (D) Immunofluorescence detection of RHAMM in RHAMM^+/+^ mouse ES cells co-localized with microtubules (acetylated tubulin; ac-tubulin) and cell-cell junctions (ZO-1). Arrows indicate co-localization with mitotic spindles, while double arrows indicate co-localization with mitotic centrosomes. Scale bar: 20 µm. (E) RHAMM^+/+^ mouse ES cells grown without feeders were fixed in 4% paraformaldehyde and stained with antibodies to either RHAMM or beta-tubulin. The arrows point out mitotic spindles that stain positive despite the lack of detergent in the fixation procedure. Addition of detergent (0.1% Triton X-100) to the methodology results in significantly better penetration of the antibodies and amplification of the detected signal. Scale bar: 10 µm.

To determine whether extracellular RHAMM is necessary for self-renewal of mouse ES cells *in vitro*, we studied two mouse ES cell-lines with mutational inactivation of one *Hmmr/*RHAMM allele (RHAMM^+/-^) along with the parental control cell-line (RHAMM^+/+^). We confirmed mutational insertion of the gene-trap (Figure 1A) and found that RHAMM expression was reduced in RHAMM^+/-^ cells, as measured by RT-PCR (Figure 1A), Western blot (Figure 1B), and fluorescence cytometry (Figure 1C). Doubling times were comparable between the cell-lines as were the proportions of cells in proliferative phases of the cell cycle, as measured by DNA content (Figure S1); however, RHAMM^+/-^ cells reached a growth plateau earlier than RHAMM^+/+^ cells as broader RHAMM^+/-^ colonies tended to undergo contact-inhibition at day 4 of culture (Figure S1). Importantly, the gene encoding RHAMM (i.e., *Hmmr*) clusters within 400bp from a gene, termed *Nudcd2*, whose product also binds to the dynein complex to localize to the centrosome [17]. This small *Hmmr-Nudcd2* cluster is conserved throughout vertebrate evolution (Figure S2). For these reasons, we confirmed by RT-PCR that the expression of *Nudcd2* was not altered in RHAMM^+/-^ mouse ES cell-lines (Figure 1A).

Next, we studied the localization of RHAMM by fluorescence cytometry. For our analyses, we included known extracellular (SSEA-1) and intracellular (TPX2) proteins as controls for the unknown localization of RHAMM; moreover, we detected RHAMM with commercial antibodies raised against either the amino- (not shown) or carboxy-terminus of the protein. Finally, as a control for specificity, we examined the protein’s localization in RHAMM^+/+^ and RHAMM^+/-^ cells, and we expected that the intensity of specific staining would be lower in the RHAMM^+/-^ cells. With these controls in place, we did not identify extracellular RHAMM, but SSEA-1 was strongly positive for both cell-lines (Figure 1C). Conversely, both RHAMM and TPX2 were detected in permeabilized cells (Figure 1C). RHAMM^+/+^ and RHAMM^+/-^ mouse ES cells contained equivalent intracellular levels of TPX2 while the levels of intracellular RHAMM were proportional to the transcript and protein expression levels detected by RT-PCR and Western blot analysis, respectively.

To validate our cytometry analyses, we examined the localization of RHAMM in permeabilized cells by confocal microscopy. We found that intracellular RHAMM co-localized with microtubules, mitotic centrosomes (double arrows), and mitotic spindles (arrows) by immunofluorescence (Figure 1D). A prior publication identified a significant fraction of putative extracellular RHAMM in human ES cells through fluorescence cytometry [6]. While no specificity controls were included in the published analysis, we were curious whether the fixation protocol commonly used for fluorescence cytometry may allow antibodies to penetrate fixed, but not detergent permeabilized, cells. In fixed cells (PFA, 4%), we found that antibodies against RHAMM, or tubulin, penetrated the cells and resulted in fluorescence detection of mitotic spindles, although not to the extent of those cells treated with detergent (Figure 1E). Penetration of antibodies following PFA fixation may help to explain the published detection of “extracellular” RHAMM [6]. However, RHAMM is an intracellular, cytoskeletal protein in mouse ES cells.

### Genomic loss of *Hmmr/*RHAMM attenuates the pluripotency of mouse ES cells

**Figure 2:**
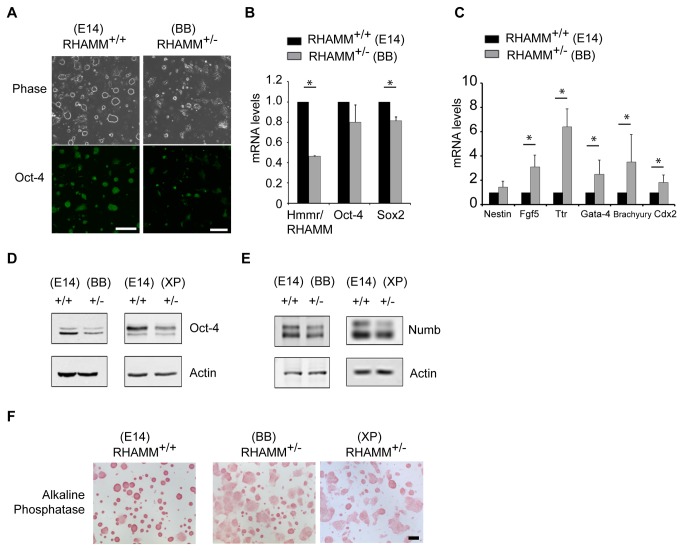
Genomic loss of RHAMM attenuates pluripotency and augments differentiation in mouse ES cells. (A) ES cell colonies are flatter and immunofluorescence detection of the pluripotency marker Oct-4 is reduced in feeder-free cultures of RHAMM^+/-^ mouse ES cells (BB0166). Scale bar: 200 µm. (B) Quantitative RT-PCR analysis of the expression levels for markers of pluripotency in RHAMM^+/-^ mouse ES cells. All expression levels were first normalized to internal GAPDH control levels, and then mRNA expression levels in RHAMM^+/-^ ES cells were normalized to the respective levels in RHAMM^+/+^ ES cells. Data are mean ± SD, n = at least duplicate experiments, * p< 0.05 for two-tailed, pairwise Student’s *t* test; p(Oct-4) = 0.07. (C) Quantitative RT-PCR analysis of the expression levels for markers of lineage differentiation in RHAMM^+/-^ mouse ES cells. All expression levels were first normalized to internal GAPDH control levels, and then mRNA expression levels in RHAMM^+/-^ ES cells were normalized to the respective levels in RHAMM^+/+^ ES cells. Data are mean ± SE, * p< 0.05 for two-tailed, pairwise Student’s *t* test, n = triplicate experiments; a fourth experiment was performed and the results are shown in Figure S3. (D) Western blot analysis of whole cell lysates revealed a marked reduction of Oct-4 protein in feeder-free RHAMM^+/-^ mouse ES cultures (BB0166 and XP0038). Actin served as a loading control. (E) Western blot analysis revealed a reduction of Numb protein in RHAMM^+/-^ mouse ES cells relative to RHAMM^+/+^ mouse ES cells, with actin serving as a loading control. Cell lysates, and loading controls, are the same as those used in Figure 1B. (F) Alkaline phosphatase staining was markedly decreased in RHAMM^+/-^ mouse ES cell colonies. Scale bar: 200 µm.

While expanding RHAMM^+/-^ mouse ES cells without feeder cells for the fluorescence cytometry and confocal microscopy analysis, we noted a change in the size and shape of mouse ES cell colonies in relation to those of RHAMM^+/+^ mouse ES cells. That is, colonies from RHAMM^+/-^ mouse ES cells were broader, flatter and less circular (Figure 2A), which implied that the genomic loss of RHAMM may attenuate the self-renewal of mouse ES cells or promote their differentiation. To address this hypothesis, we stained RHAMM^+/-^ mouse ES cell colonies with the pluripotency marker Oct-4 and found a dramatic reduction in the intensity of immunofluorescence (Figure 2A). The levels of expression of the pluripotency markers Oct-4 and Sox2 were then measured by quantitative RT-PCR and found to be reduced in RHAMM^+/-^ relative to RHAMM^+/+^ mouse ES cells (Figure 2B). Conversely, the expression of lineage markers for ectoderm (Nestin, Fgf5), endoderm (Ttr, Gata-4), and mesoderm (Brachyury), as well as the levels for a marker of the trophectoderm (Cdx2), were elevated between 2–9 fold in RHAMM^+/-^ relative to RHAMM^+/+^ mouse ES cells (Figure 2C); in one set of experiments, which was deemed an outlier and excluded from the analysis, the level of expression of lineage markers were 4-130 fold higher in RHAMM^+/-^ mouse ES cells (Figure S3). As the expression level for Oct-4 transcripts was lower in RHAMM^+/-^ cells, we confirmed this observation at the protein expression level with Western blot analysis of whole cell lysates from two redundant RHAMM^+/-^ mouse ES cell-lines in relation to the parental RHAMM^+/+^ mouse ES cells (Figure 2D). Moreover, Western blot analysis revealed a modest reduction in Numb (Figure 2E), an inhibitor of the Notch signaling pathway, in RHAMM^+/-^ mouse ES cells. We then stained the colonies for alkaline phosphatase (AP) activity, as a sensitive, specific and quantitative marker for ES cell pluripotency [18]. Consistent with the observed reduction in Sox2 and Oct-4, AP activity was reduced in large and flat, non-circular RHAMM^+/-^ mouse ES cell colonies (Figure 2F).

**Figure 3:**
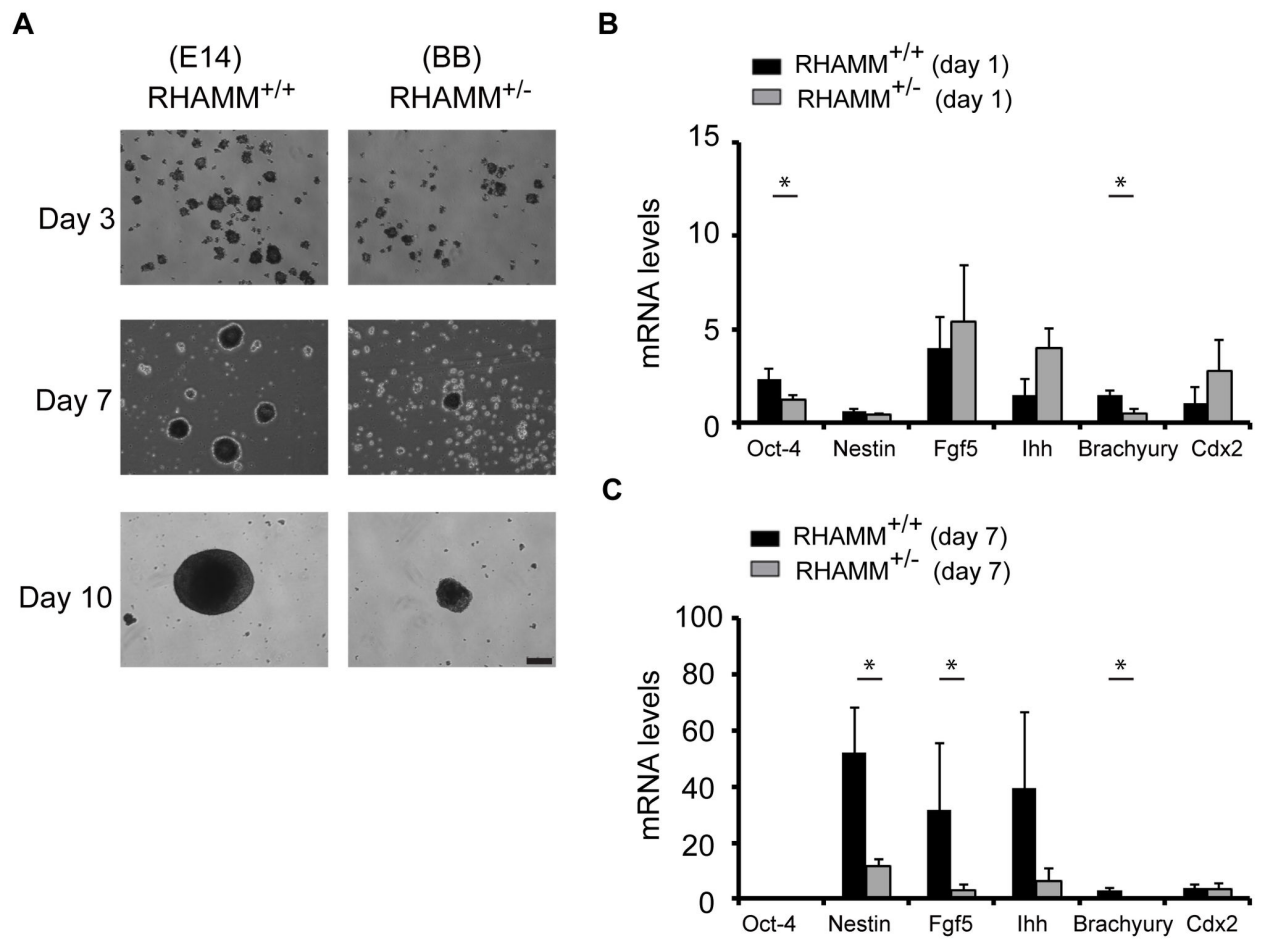
Embryoid body formation is attenuated in RHAMM^+/-^ mouse ES cells. (A) Embryoid bodies were formed at low seeding density (2 X 10^4^ per ml) with suspension cultures of RHAMM^+/+^ and RHAMM^+/-^ mouse ES cells and imaged with brightfield microscopy through the first ten days of culture. Scale bar: 200 µm. (B) Quantitative RT-PCR for the mRNA expression of lineage differentiation markers in embryoid bodies derived from RHAMM^+/+^ and RHAMM^+/-^ mouse ES cells at day 1 of culture. All expression data were first normalized to internal GAPDH control levels, and then mRNA expression levels were normalized to the respective levels in parental ES cells. Data are mean ± SE, n = triplicate experiments, * p< 0.05 for two-tailed, pairwise Student’s *t* test. (C) Quantitative RT-PCR for the mRNA expression of lineage differentiation markers in embryoid bodies derived from RHAMM^+/+^ and RHAMM^+/-^ mouse ES cells at day 7 of culture. All expression data were first normalized to internal GAPDH control levels, and then mRNA expression levels were normalized to the respective levels in parental ES cells. Data are mean ± SE, n = triplicate experiments, * p< 0.05 for two-tailed, pairwise Student’s *t* test; p(Ihh) = 0.06.

RHAMM^+/+^ and RHAMM^+/-^ mouse ES cells were next cultured in suspension on non-adherent plates to promote embryoid body formation and spontaneous differentiation toward the three lineages. We followed the differentiation potential of RHAMM^+/+^ and RHAMM^+/-^ mouse ES cells through the quantitation of embryoid body size and the expression of markers for cells derived from ectoderm, endoderm, and mesoderm lineages. RHAMM^+/+^ and RHAMM^+/-^ mouse ES cells both formed aggregates in suspension cultures, but the aggregates formed from RHAMM^+/-^ mouse ES cells were smaller than those from RHAMM^+/+^ mouse ES cells; this difference was most pronounced when ES cells were seeded in suspension at low density (2 X 10^4^ per ml, Figure 3A). Quantitative RT-PCR analysis of the expression of markers for differentiation along ecto-, endo-, and mesoderm lineages was performed on embryoid bodies isolated after one day and seven days of suspension culture. At day 1 of culture, the normalized level of expression for Oct-4 was reduced and, with the exception of brachyury, the expression of markers for differentiation along ecto-, endo-, and mesoderm lineages were slightly elevated in RHAMM^+/-^ relative to RHAMM^+/+^ mouse ES cells (Figure 3B), which is consistent with the hypothesis of precocious differentiation in RHAMM^+/-^ mouse ES cells. However, at day 7 of culture as embryoid bodies, the normalized expression levels of markers for ecto-, endo-, and mesoderm lineages were significantly reduced in RHAMM^+/-^ mouse ES cells, which we interpret as indicative of an attenuated ability to spontaneously differentiate as embryoid bodies for RHAMM^+/-^ relative to RHAMM^+/+^ mouse ES cells. Taken together, these data indicate that the genomic loss of *Hmmr*/RHAMM limits self-renewal and augments precocious differentiation in mouse ES cells.

### ERK1/2 and AURKA are barrier kinases for pluripotency that are elevated in RHAMM^+/-^ mouse ES cells.

**Figure 4:**
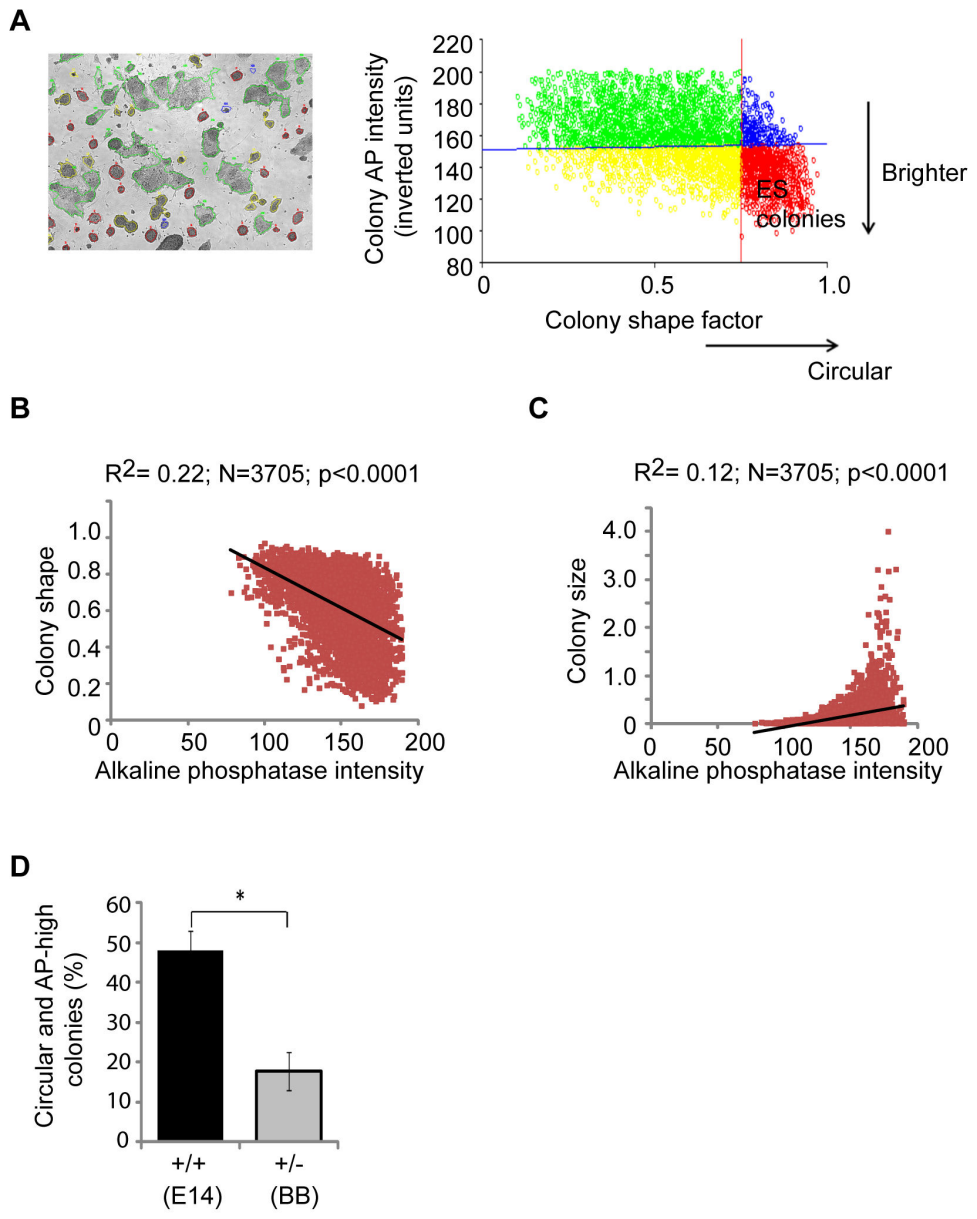
High content analysis of mouse ES cell colonies grown in the absence of feeder cells. (A) A representative image of colonies segmented by InCell developer software (GE Healthcare). First, the intensity of the alkaline phosphatase (AP) stain was inverted. Colonies were then categorized based on their inverted AP intensity and colony size and shape. Supervised analysis established cut-offs of 150 inverted units (inverted AP activity) and a shape factor of 0.75, as the best measure for mouse ES cell colonies. (B) Across all genotypes and treatments, a strong negative correlation was observed between colony shape factor and the inverse AP stain intensity. Both shape and AP activity are graphed in arbitrary units. (C) Across all genotypes and treatments, a strong positive correlation between colony size and the inverse AP stain intensity. (D) High content measurement of circular and AP-high colonies indicated a significant reduction of ES colonies in RHAMM^+/-^ cultures. Data are mean ± SD, n = 3, * p < 0.05 for two-tailed, pairwise Student’s *t* test.

For insight into a putative mechanism through which the genomic loss of RHAMM may attenuate self-renewal, we developed a high content image analysis assay to screen for small-molecules that recover pluripotency in RHAMM^+/-^ mouse ES cells. We focused our assay on the intensity of AP activity, a specific and quantitative marker for ES cell pluripotency [18], as well as the size and shape of colonies (Figure 4A). For ease of colony segmentation, the AP intensity was inverted and then correlated with either the size of the colony or the shape of the colony across all treatments and genotypes (n= 3,705). Regardless of treatment, AP activity was inversely correlated with colony size and positively correlated with circular shape factor (Figure 4B, C). The correlation between AP activity and colony shape factor was the most significant (n= 3,705; r= 0.22; *P*< 0.001), so we optimized cut-off values to accurately segment pluripotent colonies based upon these two values (i.e., inverted AP intensity < 150 units; colony shape factor > 0.75) (Figure 4A). With these settings in place, we confirmed that our assay captured the reduced pluripotency in RHAMM^+/-^ colonies (Figure 4D), and then we screened for its recovery in the presence of small-molecule inhibitors.

**Figure 5:**
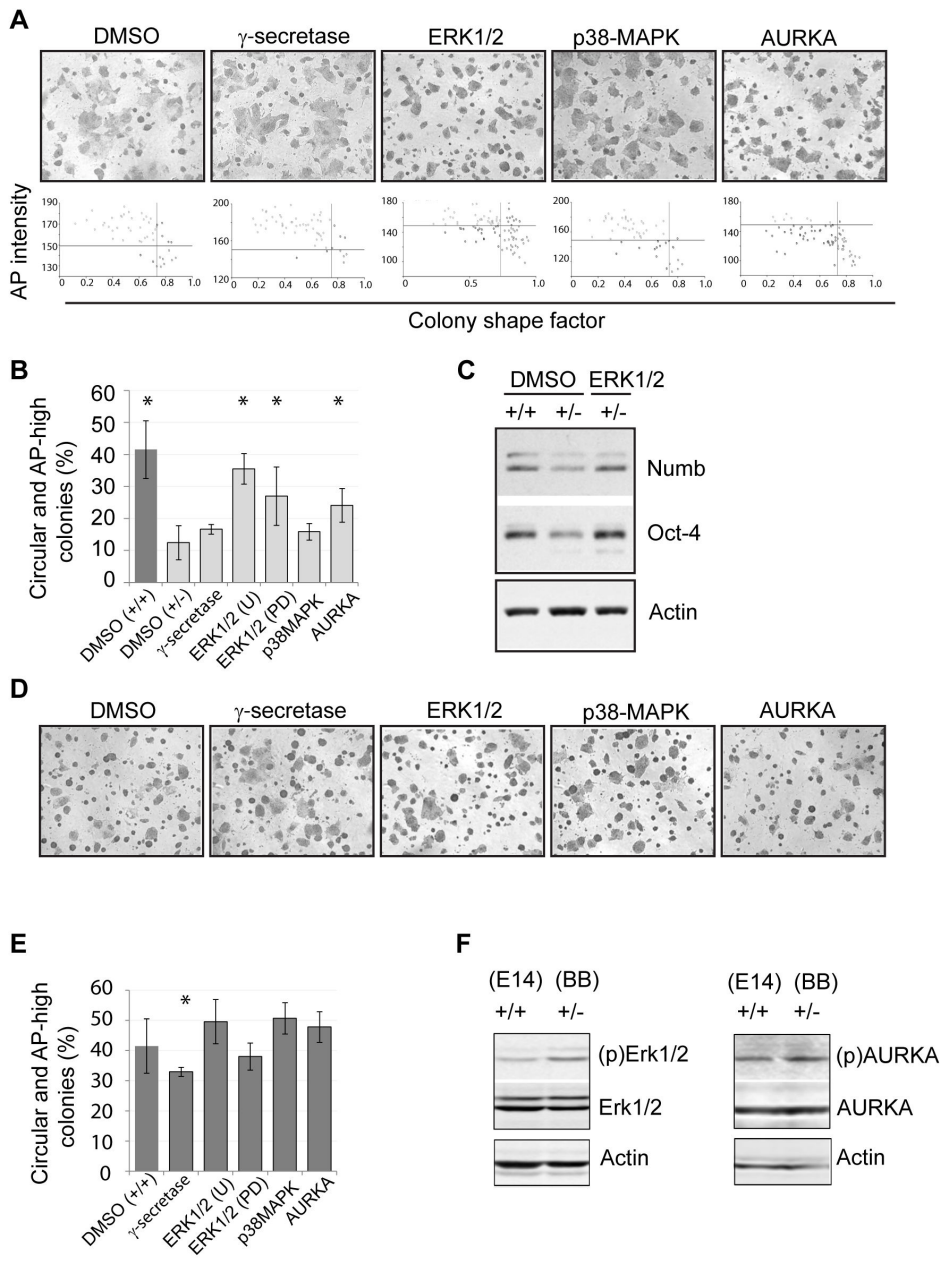
A small-molecule inhibitor screen for candidate barrier kinases. (A) RHAMM^+/-^ mouse ES cells were cultured without feeder cells in the presence of small-molecule inhibitors to the indicated kinases and enzymes for three days and colonies were then stained for alkaline phosphatase (AP) activity. Representative images were taken three days after treatment at 4 X magnification. Lower panels show the scatter distribution of colonies based on their inverted AP intensity (y axis) and shape factor (x axis). (B) High content quantification of AP staining in small-molecule inhibitor- treated RHAMM^+/-^ mouse ES cells, or DMSO-treated RHAMM^+/+^ mouse ES cells (dark bar), identified ERK1/2 and AURKA as barrier kinases against pluripotency in RHAMM^+/-^ mouse ES cells. Data are mean ± SD, n = 3, *p < 0.05 versus DMSO (+/-). (C) Following incubation with an ERK1/2 kinase inhibitor, Western blot analysis indicated marked increase in Oct-4 and Numb levels in RHAMM^+/-^ mouse ES cells and recovery to levels seen in RHAMM^+/+^ mouse ES cells. (D) Representative images of RHAMM^+/+^ mouse ES cells three days after treatment with small-molecule inhibitors to the indicated kinases and enzymes. (E) High content quantification of AP staining in RHAMM^+/+^ mouse ES cells treated with small molecule inhibitors to the indicated kinases and enzymes. Data are mean ± SD, n = 3, * p < 0.05 versus DMSO (+/+) for two-tailed, pairwise Student’s *t* test. (F) In RHAMM^+/-^ mouse ES cells, Western blot analysis indicated a marked increase in the levels of phospho (p) - ERK1/2 and phospho (p) - AURKA, which is indicative of kinase activity, but not absolute levels of the respective kinases. Actin served as a loading control.

We chose a candidate approach to identify potential kinase barriers to pluripotency that may accompany the genomic loss of RHAMM. The silencing of RHAMM in mouse and human cells has been shown to directly alter MEK1/ERK1/2 and AURKA signal transduction pathways [14,15], while RHAMM^+/-^ cells have attenuated levels of the Notch inhibitor, Numb (Figure 2E), which implies that these cells may be responsive to the inhibition of this pathway through gamma-secretase. So, RHAMM^+/-^ mouse ES cells were grown for three days in the presence of small-molecule inhibitors for AURKA, MEK/ERK1/2, or gamma-secretase; a p38-MAPK inhibitor was included as a negative control that targets a related pathway that may not be directly affected by changes in RHAMM [19,20]. In RHAMM^+/-^ cells, we found that the inhibition of the MEK/ERK1/2 pathway, with redundant small-molecule inhibitors, was sufficient to rescue colony shape and AP intensity (Figure 5A, B). Moreover, the levels of Oct-4 and Numb were recovered when RHAMM^+/-^ cells were exposed to MEK/ERK1/2 inhibitors (Figure 5C). To a lesser but still significant extent, inhibition of AURKA also recovered colony shape and AP intensity in RHAMM^+/-^ mouse ES cells (Figure 5A, B). In parallel, we performed a counter-screen against RHAMM^+/+^ mouse ES cells to determine whether the effects of small-molecule inhibitors were specific to cells with genomic loss of RHAMM. In RHAMM^+/+^ mouse ES cells, we found that only the gamma-secretase inhibitor had a significant impact on colony shape and AP intensity (Figure 5D, E). These findings suggested that the genomic loss of RHAMM alters MEK/ERK1/2 and AURKA signal transduction pathways. To investigate this, we examined whole cell lysates for the abundance of the active forms of these kinases, as well as the total kinase levels. While the total levels of the respective kinases were equivalent, we found that active forms of ERK1/2 and AURKA were both augmented in whole cell lysates from RHAMM^+/-^ mouse ES cells in relation to RHAMM^+/+^ mouse ES cells (Figure 5F). Thus, we conclude that the hemizygous loss of the cytoskeletal gene product RHAMM attenuates the self-renewal of ES cell by augmenting the activity of ERK1/2 and AURKA, which act as barrier kinases to the maintenance of pluripotency.

## Discussion

As hyaluronan (HA) is a major niche component for many stem cells, the cell-surface receptors responsible for HA-mediated signal transduction are critical for stem cell homeostasis. Here, we present two major findings. First, we find that RHAMM does not localize to the cell-surface in murine ES cells but rather is a cytoskeletal and mitotic spindle protein. Second, we find that RHAMM does provide necessary cues for the maintenance of stem cell pluripotency, but it does so through the regulation of signal transduction pathways at microtubules, specifically ERK1/2 and AURKA.

RHAMM was originally named as an extracellular secreted protein [8]; however, a mechanism that explains its secretion and putative extracellular functions is unknown. In fact, the primary structure of RHAMM is hydrophilic and lacks hydrophobic residues or a defined signal peptide that would enable secretion by classical pathways. As extracellular RHAMM is generally studied within cancer cells, or during wounding and inflammation, the protein may be released upon cell death during these pathological states [21]. However, it has been suggested that cell-surface RHAMM may play a physiological role during the self-renewal of human ES cells [6]. In our analyses of the localization of RHAMM in murine ES cells, we included positive controls for cell-surface and intracellular localization, and we examined cell-lines with known changes in the expression of RHAMM (i.e., wild-type and heterozygous expression) with the expectation that non-specific staining would be uniform across these cells; in human cells, an equivalent specificity control would be the examination of cells treated with siRNA specific to RHAMM, or with non-targeting scrambled siRNA controls. Moreover, we ensured that our fixation procedure did not allow antibodies to penetrate the cells, which would result in the false positive identification of RHAMM on the cell-surface. After including these controls, we were not able to localize RHAMM to the cell-surface of mouse ES cells and instead found that RHAMM is a cytoskeletal protein in these cells.

While it is likely that RHAMM and other intracellular proteins are released to the extracellular milieu with the cellular death that accompanies cancer, inflammation, and wounding, multiple lines of evidence suggest that RHAMM is not a cell-surface receptor under physiological conditions. First, the primary structure of RHAMM is almost entirely hydrophilic and lacks a defined signal peptide. Second, the basic C-terminus region of RHAMM that binds to hyaluronic acid *in vitro* [22,23] does not share homology with other defined hyaluronan receptors (i.e., CD44 and link proteins). In fact, the C-terminus is a conserved, basic leucine zipper motif, which directs the protein to the centrosome and plays a key role during mitotic spindle assembly [11,12,24]. Moreover, the association between the basic C-terminus of RHAMM and hyaluronic acid is not specific, as this region of RHAMM also binds heparin, and is entirely ionic in nature [22,23]. Third, the expression of RHAMM is strictly regulated through the cell cycle in tissue cultured cells [25,26], and RHAMM is detected in proliferative human tissues, such as the testes and immune cells, which is consistent with its conserved role during mitosis. Indeed, the phenotype of *Hmmr-*null animals [27] (i.e., fertility defects) supports a critical role for the protein in meiotic and mitotic divisions. Fourth, the *HMMR* gene is found adjacent to *NUDCD2* (within 400 base-pairs) in *Xenopus* , marsupials, chickens, rodents, and primates (Figure S2). Both genes encode cytoskeletal and centrosome proteins that interact with dynein and play key roles in mitosis [11,17,25]. Finally, the results of a number of unbiased screens in humans and *Xenopus* cells demonstrate that RHAMM is a microtubule and mitotic spindle protein [24,28–34].

While RHAMM is not on the cell-surface in mouse ES cells, we found that its hemizygous loss attenuated the pluripotency of these cells grown *in vitro*. However, RHAMM^-/-^ animals have been described in the literature, and these animals are viable, lack gross abnormalities, but display fertility defects [27]. These abnormalities are consistent with those described for other spindle assembly factors. Knockout mice for nine of these factors (as outlined in [35]) have been described and the germline disruption of four of these factors causes embryonic lethality (*Numa, Nusap, Tacc3, Tpx2*), two cause no phenotype (*Tacc2, Astrin*), and three display fertility defects (*Hmmr, Hurp, Tektin2*) [36]. The mild phenotype in RHAMM^-/-^ animals, and others, suggests some redundancy of function with these gene products. It is important to note, however, that RHAMM^-/-^ mice retain exons 1–7 and 17–18 of the *Hmmr* gene [27], which encode the conserved microtubule binding domain at the N-terminus and the C-terminal bZIP motif. In these animals, it is expected that the C-terminal bZIP domain is disrupted through a frame shift but the expression of an N-terminal 920 bp mRNA is retained [27]. The RHAMM^+/-^ mouse ES cells described herein have complete mutational inactivation of the *Hmmr* gene downstream from exon 3 or exon 7, respectively.

By using a genomic approach to study the reduced expression of RHAMM, as opposed to silencing expression through transient transfection of siRNA, we were able to assess effects on ES cell pluripotency in redundant RHAMM^+/-^ cell-lines without the confounding issues of transient and inconsistent loss of expression. The stable loss of RHAMM expression also enabled us to perform a small-molecule screen for the recovery of pluripotency in these cells. This screen identified ERK1/2 and AURKA, but not p38-MAPK, as kinases that function as barriers to pluripotency with the loss of RHAMM.

Together with recent published literature, our findings suggest that the maintenance of ES cell pluripotency is heavily reliant upon microtubule-associated signal transduction pathways. That is, we find that the loss of RHAMM augments MEK/ERK1/2 activity and the inhibition of this activity is sufficient to restore pluripotency. RHAMM can directly bind to and regulate ERK1/2 activity at microtubules [15], and the activation of the ERK1/2 pathway may be responsive to the levels of leukemia-inhibitory factor 1 (LIF1) - induced STAT3 phosphorylation, which prevents the differentiation of mouse ES cells downstream [37].

RHAMM also complexes with TPX2 and regulates AURKA signaling [12,14,25,38]. Consistent with this function, we found slight elevation of active AURKA in whole cell lysates from RHAMM^+/-^ mouse ES cells, and incubation with a small-molecule AURKA inhibitor was sufficient to recover colony shape and AP intensity in these cells, which implicates AURKA as a barrier to pluripotency. However, the inhibitor did not affect RHAMM^+/+^ mouse ES cells, which suggests that inhibition of AURKA affects self-renewal in a genotype-dependent manner. In fact, a recent double blind screen of 244 inhibitors identified AURKA as one of three barrier kinases to reprogramming and pluripotency [39]; a small-molecule AURKA inhibitor potently enhanced induced pluripotent stem (iPS) cell generation [39], which is similar to our finding that the same AURKA inhibitor restored pluripotency of RHAMM^+/-^ mouse ES cells. However, an alternate screen of 104 ES cell-associated phosphoregulators found that the same AURKA inhibitor caused ES cells and iPS cells to differentiate [40]. These conflicting results [39,40] may be reconciled by our finding that AURKA activity is critical to mouse ES cell self-renewal, but that AURKA inhibitors affect pluripotency in a genotype-dependent manner.

In conclusion, RHAMM is an intracellular cytoskeletal protein that modulates ERK1/2 and AURKA signal transduction at microtubules, which can act as barriers to mESC pluripotency. RHAMM is not found on the cell-surface of mouse ES cells, and future studies of hyaluronan receptors during the self-renewal of ES cell will best focus on alternate cell-surface hyaluronan receptors, such as CD44 [5].

## Supporting Information

Figure S1 **RHAMM^+/-^ and RHAMM^+/+^ mouse ES cells do not differ in progression through the cell cycle.** (A) Cell proliferation rate was not significantly different between RHAMM^+/-^ and RHAMM^+/+^ mouse ES cells grown in the absence of feeder cells. Doubling times were determined as outlined in the methods section, and are tabulated below the graph. The growth of RHAMM^+/-^ mouse ES cells started to plateau at day 4, which is attributed to contact inhibition due to the broader colony structure. (B) Flow cytometry analysis of DNA content in RHAMM^+/-^ and RHAMM^+/+^ mouse ES cells reveals equivalent proportions of cells in the proliferative phases of the cell cycle at day 3 of culture. Mean (standard deviation) values for the fractions identified in respective cell cycle phases are tabulated below the cytometry profiles.
(TIF)

Figure S2 **Conservation of *HMMR* / RHAMM - *NUDCD2* gene cluster throughout vertebrate evolution.** In fish, *NUDCD2* is within a small cluster with the gamma-aminobutyric acid (GABA) receptors. In vertebrates, however, *CCNG1-NUDCD2-HMMR-MAT2B* form a small, separate cluster proximal to the GABA receptor cluster. Data was obtained from UCSC genome browser by searching for the location of *HMMR* within genomes from human (*Homo sapiens*, chromosome 5), primate ( *Pan* *troglodytes* , chromosome 5), rodent (*Mus musculus*, chromosome 11), canine ( *Canis* *lupus* *familiaris*, chromosome 4), platypus ( *Ornithorhynchus* *anatinus* , chromosome X1), bird (*Gallus gallus*, chromosome 13 and *Taeniopygia* *guttata* , chromosome 13), frog and lizard ( *Xenopus* *tropicalis* , scaffold 3; *Anolis* *carolinensis* , chromosome 1), and fish (*Danio rerio*, chromosome 14).
(TIF)

Figure S3 **Expression levels for differentiation markers are elevated in RHAMM^+/-^ mouse ES cells.** Quantitative RT-PCR analysis of the expression levels for markers of lineage differentiation in RHAMM^+/-^ mouse ES cells. All expression levels were first normalized to internal GAPDH control levels, and then mRNA expression levels in RHAMM^+/-^ ES cells were normalized to the respective levels in RHAMM^+/+^ ES cells.
(TIF)

Table S1 **List of primers used for RT-PCR and qRT-PCR.**
(DOCX)

Table S2 **List of antibodies used for immunostaining.**
(DOCX)
