# Supplementary figures and images for: The Cytoskeletal Protein RHAMM and ERK1/2 Activity Maintain the Pluripotency of Murine Embryonic Stem Cells
Source: PLoS One. 2013 Sep 3;8(9):e73548. doi: 10.1371/journal.pone.0073548 (PMC3760809; doi:10.1371/journal.pone.0073548)

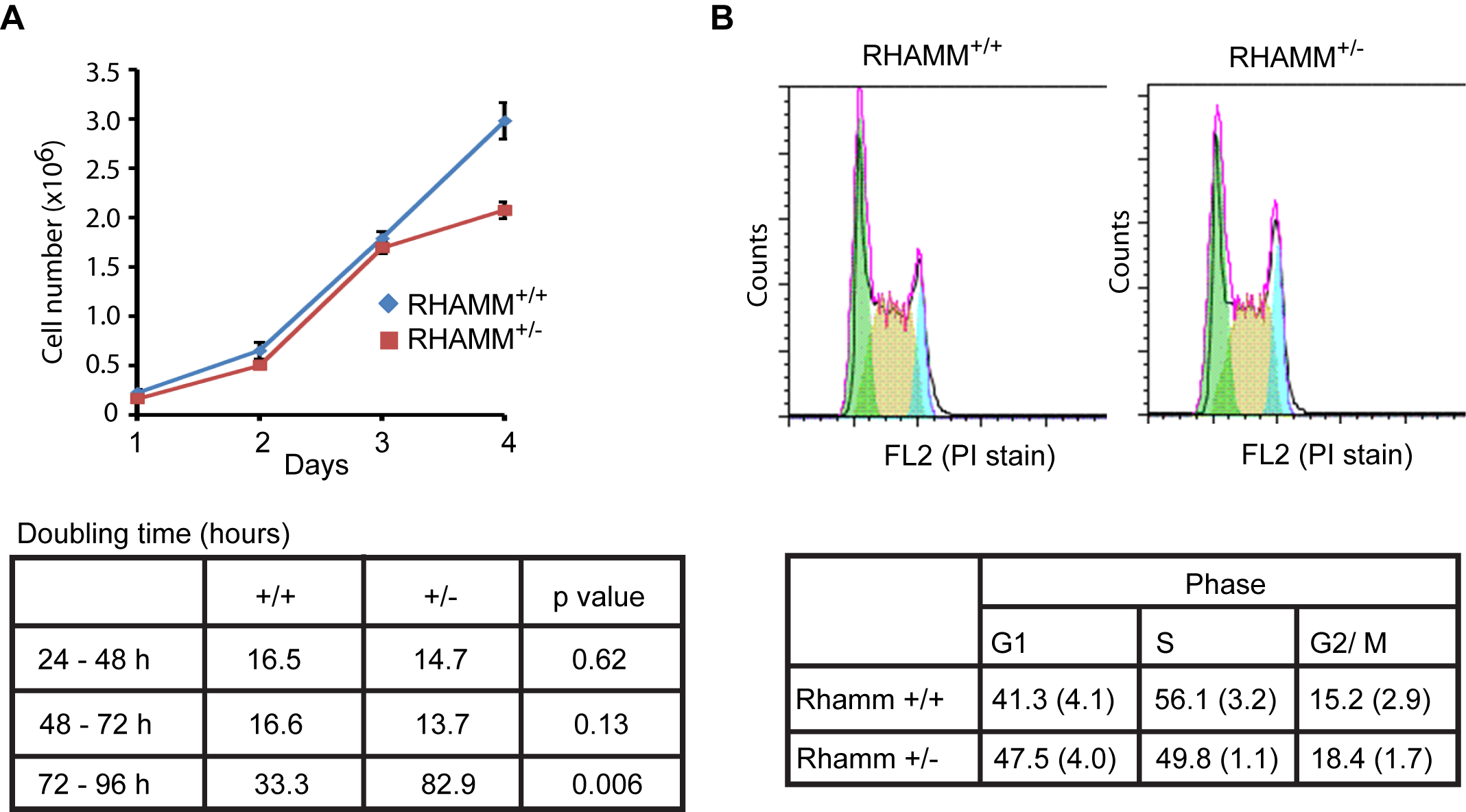

Supplement: Figure S1 — RHAMM+/- and RHAMM+/+ mouse ES cells do not differ in progression through the cell cycle. (A) Cell proliferation rate was not significantly different between RHAMM+/- and RHAMM+/+ mouse ES cells grown in the absence of feeder cells. Doubling times were determined as outlined in the methods section, and are tabulated below the graph. The growth of RHAMM+/- mouse ES cells started to plateau at day 4, which is attributed to contact inhibition due to the broader colony structure. (B) Flow cytometry analysis of DNA content in RHAMM+/- and RHAMM+/+ mouse ES cells reveals equivalent proportions of cells in the proliferative phases of the cell cycle at day 3 of culture. Mean (standard deviation) values for the fractions identified in respective cell cycle phases are tabulated below the cytometry profiles. (TIF) [file pone.0073548.s001.tif]

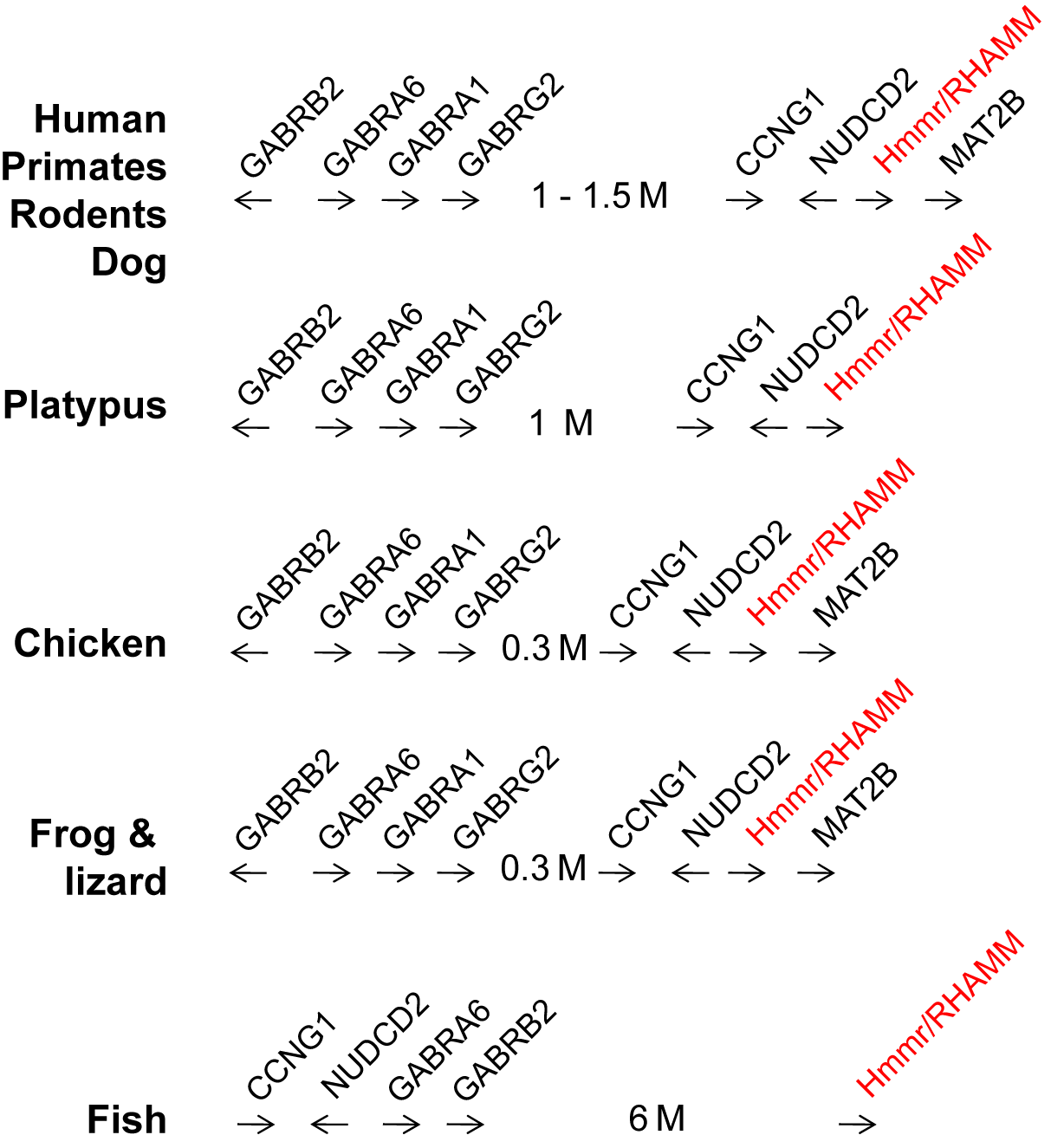

Supplement: Figure S2 — Conservation of HMMR / RHAMM - NUDCD2 gene cluster throughout vertebrate evolution. In fish, NUDCD2 is within a small cluster with the gamma-aminobutyric acid (GABA) receptors. In vertebrates, however, CCNG1-NUDCD2-HMMR-MAT2B form a small, separate cluster proximal to the GABA receptor cluster. Data was obtained from UCSC genome browser by searching for the location of HMMR within genomes from human (Homo sapiens, chromosome 5), primate ( Pan troglodytes , chromosome 5), rodent (Mus musculus, chromosome 11), canine ( Canis lupus familiaris, chromosome 4), platypus ( Ornithorhynchus anatinus , chromosome X1), bird (Gallus gallus, chromosome 13 and Taeniopygia guttata , chromosome 13), frog and lizard ( Xenopus tropicalis , scaffold 3; Anolis carolinensis , chromosome 1), and fish (Danio rerio, chromosome 14). (TIF) [file pone.0073548.s002.tif]

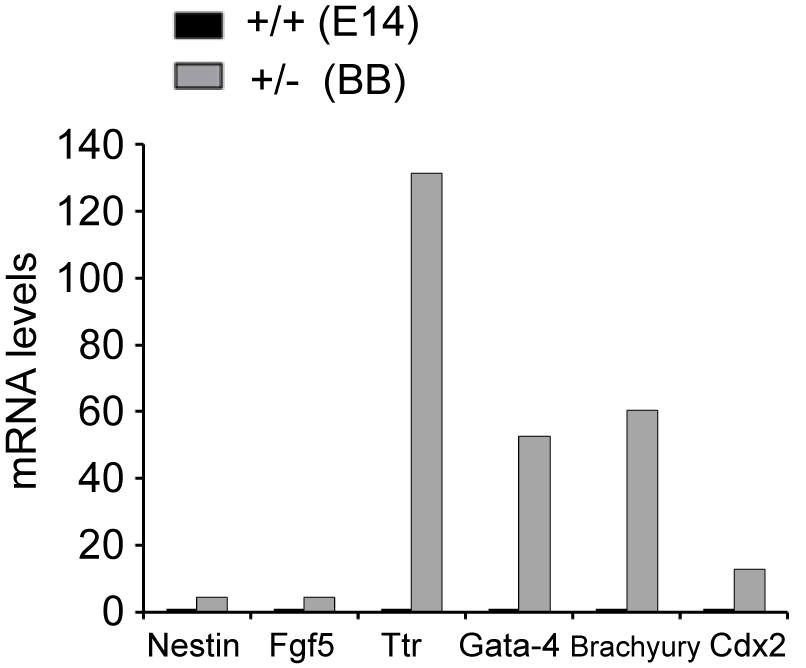

Supplement: Figure S3 — Expression levels for differentiation markers are elevated in RHAMM+/- mouse ES cells. Quantitative RT-PCR analysis of the expression levels for markers of lineage differentiation in RHAMM+/- mouse ES cells. All expression levels were first normalized to internal GAPDH control levels, and then mRNA expression levels in RHAMM+/- ES cells were normalized to the respective levels in RHAMM+/+ ES cells. (TIF) [file pone.0073548.s003.tif]
