# Supplementary material for: The Cytoskeletal Protein RHAMM and ERK1/2 Activity Maintain the Pluripotency of Murine Embryonic Stem Cells
Source: PLoS One. 2013 Sep 3;8(9):e73548. doi: 10.1371/journal.pone.0073548 (PMC3760809; doi:10.1371/journal.pone.0073548)
Supplement: Table S1 — List of primers used for RT-PCR and qRT-PCR. (DOCX) [file pone.0073548.s004.docx]

**Table S1**. List of primers used for RT-PCR and qRT-PCR

| Gene/  mRNA | Cycles (temp) | Forward primer (5’-3’) | Reverse primer (5’-3’) | Size (bp) |
| --- | --- | --- | --- | --- |
| *Hmmr* Ex1/11 | 35  (55°C) | ATTCAATGACCCTTCGGGTTGTGC | TTTCACTCTGCTCCTCCTTCTGCT | 1159 |
| *Hmmr* Ex5/11 | 35  (55°C) | GCAAAGCTCAATGCAGCAGTCAG | TTTCACTCTGCTCCTCCTTCTGCT | 837 |
| *Hmmr* Gene trap | 35  (55°C) | GCAAAGCTCAATGCAGCAGTCAG | ATTCAGGCTGCGCAACTGTTGGG | 544 |
| *Nudc d2* | 30 (55°C) | AGGTGTTCATTGAAGTCCAGGTGC | AAGGTTTGAGAAATCTGGTCCGCC | 386 |
| ***Hmmr*** | 40  (60^o^C) | AACAACTGGATGCCTTTGAAGCCG | AGCCTTGGAAGGGTCAAAGTGTCT | 287 |
| ***Sox2*** | 40  (60^o^C) | ATGGACAGCTACGCGCAC | CGAGCCGTTCATGTAGGTCTG | 192 |
| ***Oct 3/4*** | 40  (60^o^C) | CCAATCAGCTTGGGCTAGAG | CCTGGGAAAGGTGTCCTGTA | 129 |
| ***Nestin*** | 40  (60^o^C) | CTGCAGGCCACTGAAAAGT | TTCCAGGATCTGAGCGATCT | 89 |
| ***Fgf5*** | 40  (60^o^C) | TGTGTCTCAGGGGATTGTAGG | AGCTGTTTTCTTGGAATCTCTCC | 137 |
| ***Ttr*** | 40  (60^o^C) | TCGCCACTACACCATCGCA | ATGGGATGCTACTGCTTTGGCA | 129 |
| ***Gata-4*** | 40  (60^o^C) | GAAAACGGAAGCCCAAGAACC | TGCTGTGCCCATAGTGAGATGAC | 212 |
| ***Ihh*** | 40  (60^o^C) | TCACCCCCAACTACAATCCCG | GGCCATCTTCATCCCAGCCT | 171 |
| ***Brachyury*** | 40  (60^o^C) | TCACCAACAAGCTCAATGGAGG | GGTCTCGGGAAAGCAGTGGC | 128 |
| ***Cdx2*** | 40  (60^o^C) | ACGCTGCGAGAATCCTCAGAAG | CTGGCAGGAAGAGTCGGAAT | 166 |
| ***Gapdh*** | 40  (60^o^C) | AGGTCGGTGTGAACGGATTTG | TGTAGACCATGTAGTTGAGGTCA | 123 |
| *Gapdh* | 28 (55°C) | CTCGTCTCATAGACAAGATGGTGAAG | AGACTCCACGACATACTCAGCACC | 305 |

Primers in bold were used for qRT-PCR
