# Supplementary material for: The Cytoskeletal Protein RHAMM and ERK1/2 Activity Maintain the Pluripotency of Murine Embryonic Stem Cells
Source: PLoS One. 2013 Sep 3;8(9):e73548. doi: 10.1371/journal.pone.0073548 (PMC3760809; doi:10.1371/journal.pone.0073548)
Supplement: Table S2 — List of antibodies used for immunostaining. (DOCX) [file pone.0073548.s005.docx]

**Table S2**. List of antibodies used for immunostaining.

| Antibodies | Dilution | Host | Source |
| --- | --- | --- | --- |
| Oct3/4 | 1:100 | mouse | StemCell Technology 01550 |
| RHAMM | 1:500 | rabbit | Epitomics 5129-1 |
| ZO-1 | 1:100 | mouse | Zymed (invitrogen) 33-9100 |
| Beta-tubulin-647 | 1:500 | rabbit | Cell signaling 3624 |
| acetylated tubulin | 1:500 | mouse | Sigma T7451 |
